# Multispectral singlet oxygen and photosensitizer luminescence dosimeter for continuous photodynamic therapy dose assessment during treatment

**DOI:** 10.1117/1.JBO.25.6.063810

**Published:** 2020-03-13

**Authors:** Tobias J. Moritz, Youbo Zhao, Michael F. Hinds, Jason R. Gunn, Jennifer R. Shell, Brian W. Pogue, Steven J. Davis

**Affiliations:** a Physical Sciences Inc., Andover, Massachusetts, United States; b Thayer School of Engineering at Dartmouth, Hanover, New Hampshire, United States

**Keywords:** photodynamic therapy, singlet oxygen, dose, tumor regrowth, spectroscopy

## Abstract

**Significance:** Photodynamic therapy (PDT) involves complex light-drug-pathophysiology interactions that can be affected by multiple parameters and often leads to large variations in treatment outcome from patient to patient. Direct PDT dosimetry technologies have been sought to optimize the control variables (e.g., light dose, drug administration, tissue oxygenation, and patient conditioning) for best patient outcomes. In comparison, singlet oxygen (O21) dosimetry has been tested in various forms to provide an accurate and perhaps comprehensive prediction of the treatment efficacy.

**Aim:** We discuss an advanced version of this approach provided by a noninvasive, continuous wave dosimeter that can measure near-infrared spectrally resolved luminescence of both photosensitizer (PS) and O21 generated during PDT cancer treatment.

**Approach:** This dosimetry technology uses an amplified, high quantum efficiency InGaAs detector with spectroscopic decomposition during the light treatment to continuously extract the maximum signal of O21 phosphorescence while suppressing the strong PS luminescence background by spectrally fitting the data points across nine narrow band wavelengths. O21 and PS luminescence signals were measured *in vivo* in FaDu xenograft tumors grown in mice during PDT treatment using Verteporfin as the PS and a continuous laser treatment at 690 nm wavelength.

**Results:** A cohort of 19 mice was used and observations indicate that the tumor growth rate inhibition showed a stronger correlation with O21 than with just the PS signal.

**Conclusions:** These results suggest that O21 measurement may be a more direct dosimeter of PDT damage, and it has potential value as a definitive diagnostic for PDT treatment, especially with spectral separation of the background luminescence and online estimation of the PS concentration.

## 1 Introduction

Photodynamic therapy (PDT) is a nonionizing light-activated chemotherapy.^1^^,^^2^ It has been clinically investigated for treatment of a variety of cancers including oral, bladder, brain, skin, esophageal, and other cancers.^3^^–^^5^ For skin cancer where light delivery is easily achievable, the advantages of PDT have been extensively demonstrated.^6^^–^^8^ During PDT, a combination of light and selective photosensitizers (PS) is administered. Upon light illumination, the PS is initially excited to a singlet state that emits prompt fluorescence. However, a large fraction of singlet state PS molecules transition to a metastable triplet state via an intrasystem crossing process. The triplet state PS is collisionally quenched by ground state oxygen molecules that populate singlet state oxygen (0.98 eV above triplet ground state) within the treated tumor. The generated singlet oxygen (O21) is responsible for the destruction of tumor cells through: (a) direct oxidative damage to cell membranes and organelles^9^ and (b) vascular damage and constriction that starves the tumor of nutrients.^10^ PDT efficacy strongly depends on the amount of O21 produced during the treatment, which in turn, is influenced by multiple parameters: the PS concentration in the tumor, intracellular localization, treatment light intensity, total light dose delivered (fluence), and tumor oxygenation. Differences in these parameters can lead to large variations in the treatment outcome of individual patients.

PDT dosimetry capable of quantifying O21 production during treatment would be a valuable tool to guide optimization of clinical variables. Accumulating evidence indicates that the variations in treatment outcome have a strong correlation with the amount of O21 produced within the tumor.^11^^–^^16^ Several methodologies have been investigated for PDT dosimetry.^17^^–^^19^ In principle, an explicit dosimeter that quantifies all the contributing parameters would be ideal. This would require measurements of dynamic drug and oxygen concentrations that entered into theoretical models.^11^^,^^12^^,^^20^ Extensive *in vivo* studies and very promising results on this topic have been published.^11^^–^^13^ For example, using an explicit model, one study calculated O21 concentrations at different radial distances from the excited benzoporphyrin derivative monoacid ring A (BPD). These calculations have the potential to be used to determine pretreatment patient-specific parameters to predict and optimize personalized PDT outcome. Macroscopic models have also been used^14^^,^^15^ to calculate concentrations of reactive oxygen species as a means to predict Photofrin-PDT outcome in mice.^16^ A combination of Monte–Carlo simulations and measured optical tissue properties was used to calculate the light fluence during PDT treatment. The results of this study demonstrated a correlation between O21 and the cure index and concluded that O21 is a potential quantity that can be used to predict PDT treatment outcome. However, there are still challenges in the clinical implementation of this approach, as multiple probes are needed to quantify all the contributing parameters and some of the measurements are invasive.^19^

Alternatively, a nonintrusive dosimeter that measures both the prompt PS fluorescence and the longer lived O21 phosphorescence may be considered. It is known that singlet state oxygen emits spectrally defined phosphorescence centered at ∼1270  nm. This provides an opportunity to directly measure O21 with noninvasive optical detection methods. Several groups have demonstrated near-infrared (NIR) O21 dosimetry with a range of experimental systems.^21^^–^^30^ An extensive summary of these definitive dosimetry approaches can be found in Ref. 19.

In tissue, O21 is severely quenched, and optical detection of the phosphorescence signature is very challenging with limited signal-to-noise ratio (SNR). Moreover, the presence of strong PS luminescence background that overlaps the O21 phosphorescence further complicates the measurement. Previously reported definitive O21 dosimeters used combined spectral and temporal discriminations to extract O21 from PS background. For example, previous work used square wave diode laser pulses to excite the PS, produce the singlet oxygen signal, and to extract the longer-lived O21 phosphorescence signal from the PS luminescence.^21^^–^^24^^,^^30^ This was accomplished by incorporating gated detection and observing the O21 signal after the termination of the laser pulse. With this approach, we previously observed a positive tumor regression correlation with O21 measured during PDT treatment of tumor-laden rats.^21^ Others have also evaluated the time-gated approach using pulsed Nd:YAG lasers for O21 detection and demonstrated its value and improved SNR in PDT studies.^18^^,^^27^^,^^28^^,^^31^ These temporal discrimination methodologies require the use of short-pulsed light to excite the PS, and the signal is observed immediately after the termination of the light source when the short-lived PS fluorescence has decayed and the longer-lived O21 phosphorescence is still observable. However, many preclinical and clinically approved PDT protocols use a continuous wave (cw) laser or lamp source, where such a time-resolved approach is not applicable.

In this work, we describe the development of a cw, optically based prototype PS and O21 dosimeter and its initial application to an *in vivo* study of tumor laden mice. The goal of this study was to demonstrate the feasibility of PS/O12 dosimetry where the detected PS luminescence and the O21 phosphorescence are produced directly by the cw treatment laser. As described below, this was enabled by spectral fitting processes that separated the PS luminescence from the O21 phosphorescence. Correlations of tumor growth inhibition to both PS and O21 measurements are presented to demonstrate the capability of this cw PDT dosimetry approach.

## 2 Methodology

### 2.1 Experimental Setup

**Fig. 1:**
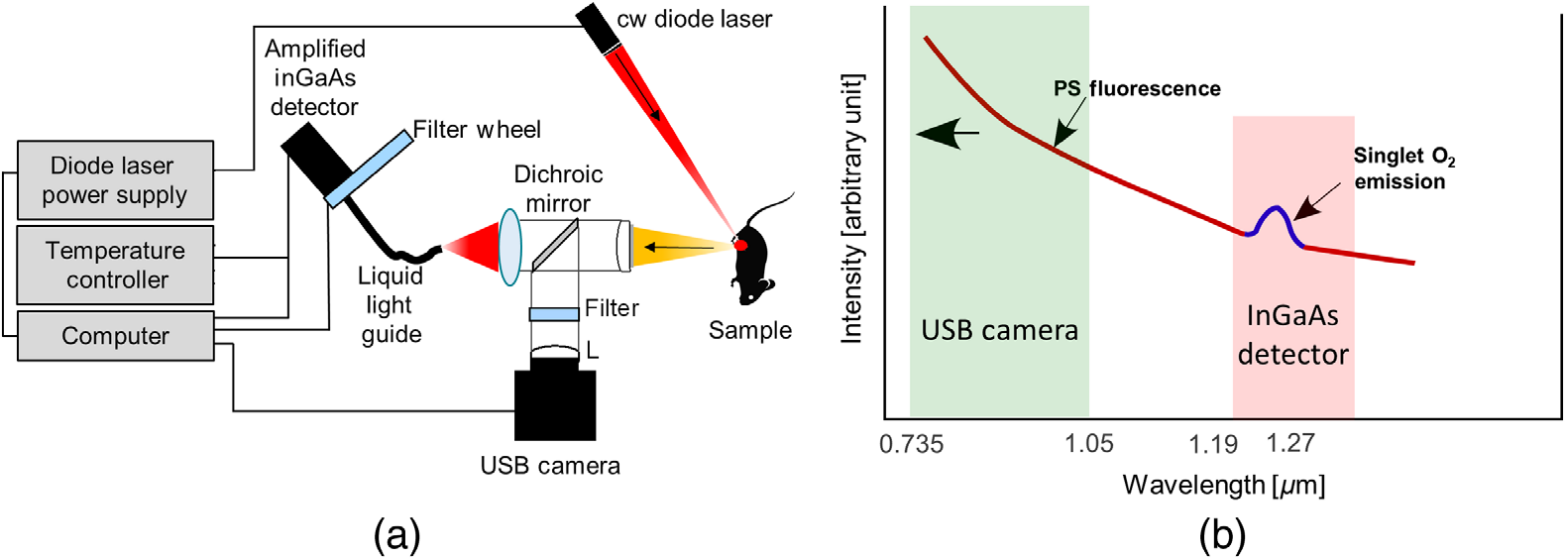
Experimental setup: (a) schematic of the prototype instrument and (b) conceptual diagram illustrating PS and O21 luminescence spectra and the two wavelength regions detected by separate detection channels.

Figure 1(a) shows a schematic setup of the prototype PS/O21 dosimeter. The system has two optical detection channels: (a) in the 735- to 1000-nm spectral range and (b) in the 1190- to 1330-nm spectral range. The short wavelength signal is produced by the PS fluorescence and is detected by a Si-CCD USB camera. The long wavelength range measures the spectrum of the combined PS luminescence and O21 phosphorescence signal that is collected by a liquid light guide and detected by a thermoelectrically cooled InGaAs detector (59-141, Edmund Optics) in combination with an automated filter wheel (filters centered at: 1193, 1222, 1250, 1261, 1271, 1283, 1291, 1315, and 1330 nm). Figure 1(b) is a diagram illustrating the spectral regions measured by both the USB camera and the InGaAs detector. The emission from the PS (indicated in red) spans from the visible spectral region (λ<735  nm not shown) all the way to the NIR. The O21 spectrum (indicated in blue) has a peak around 1270 nm that is superimposed onto the broadband PS background. The signal collected by the USB camera (green box, λ<1050  nm) only contains emission from the PS. The main purpose of the USB camera is to track the photobleaching of the PS fluorescence background.

This study used a cw 690-nm diode laser (AOC-LD-1 and AOC-TC-1, Applied Optronics Corporation) as a treatment source for the PDT studies. We note that this laser source is only needed for the treatment, and it is not a component of the dosimeter system. The dosimeter passively collects the optical emissions produced by the PDT laser. Therefore, the dosimeter can be used in all types of PDT treatments with no restriction in the selection of PS and laser source.

We used a solid-state InGaAs detector to monitor both the PS and O21 luminescence. The output of the detector was amplified with a low noise voltage preamplifier (SR560, Stanford Research Systems). In previous, pulsed dosimeter studies, we used near-IR sensitive photomultiplier tubes to provide sufficiently high bandwidth to temporally discriminate the slower decaying O21 phosphorescence from the short-lived PS fluorescence. However, the cw approach we describe here relaxes the high bandwidth detection requirement and provides 100% duty cycle for the detector. The challenge is to discriminate the singlet oxygen phosphorescence from the much more intense PS luminescence using only spectral discrimination.

### 2.2 Data Acquisition and Processing to Extract Singlet Oxygen Signal

**Fig. 2:**
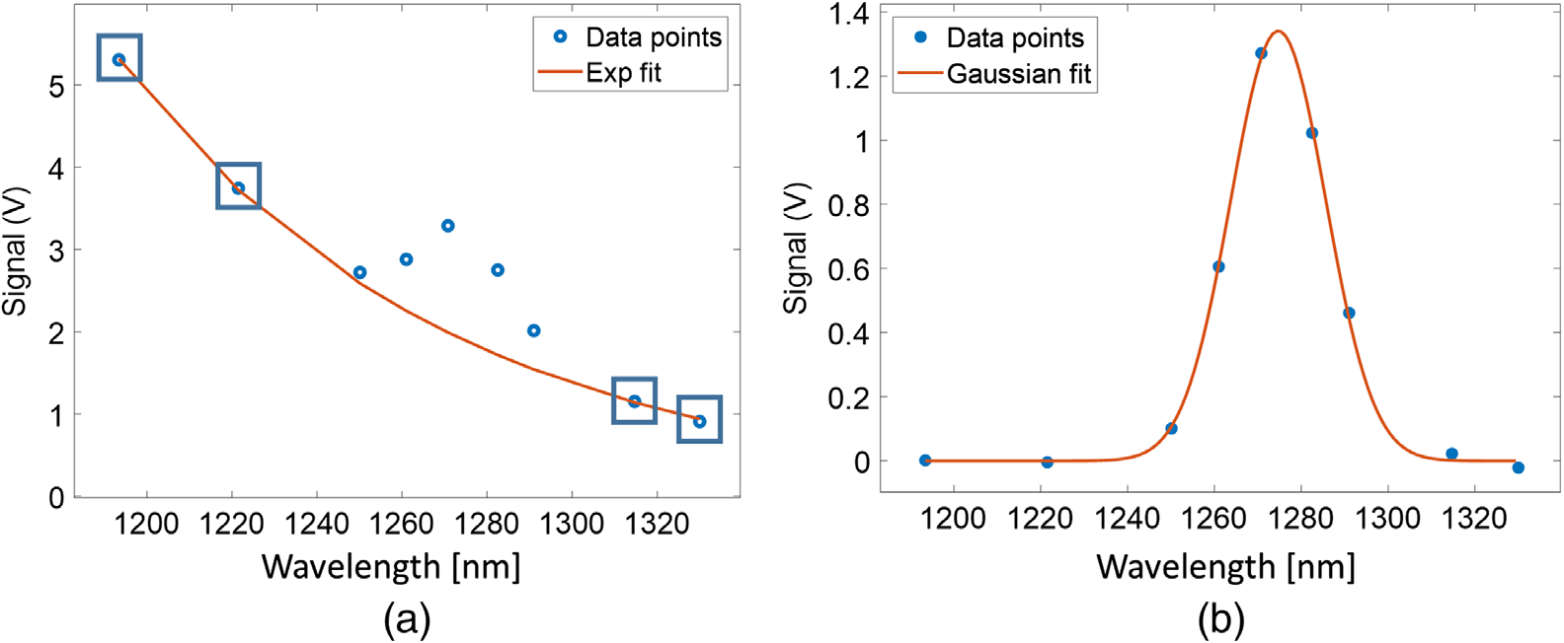
Method to extract singlet oxygen signal: (a) determination of PS (benzoporphyrin derivative, BPD) luminescence background based on exponential fitting (red curve) to the four out of band spectral data points indicated by the blue squares. (b) Gaussian fitting (red curve) to the PS fluroescence background subracted data points (blue dots), which depicts the extracted singlet oxygen (O21) signal.

The PS/O21 spectra were measured by observing the optical emissions through the nine bandpass filters listed in Sec. 2.1. A typical spectrum from a 10-μM BPD solution in methanol is shown in Fig. 2(a). These nine spectral points sampled the combined light emission spectrum of PS and O21. The acquisition time per filter position was 5 s for the InGaAs detector. Since PS luminescence may change during the measurement due to photobleaching, it would introduce artifacts to the spectral shape if not monitored throughout the PDT treatment. To account for this effect, the USB camera was synchronized with the filter wheel and used to capture an image for each filter measurement to track the temporal changes of the PS luminescence signal during the entire measurement sequence. The intensities of the USB images corresponding to the temporal coordinate of each filter were used to correct the contributions of the PS bleaching to the measured PS/O21 spectrum. Finally, the intensity responses of the system at all filter wavelengths were calibrated using a black body radiation source (SR-2-33, CI Systems).

As shown in Fig. 2(a), to extract the singlet oxygen signal from the PS background, an exponential fit was applied to four data points (filter positions: 1193, 1222, 1315, and 1330 nm, blue boxes) outside the spectral range of the O21 signal. The resulting fit (red solid line) was subtracted from the (PS+O21) spectrum in Fig. 2(a), resulting in a background (PS luminescence) subtracted O21 signal [Fig. 2(b)]. PS values were determined by summing the intensity values of the exponential fit [Fig. 2(a)] for each filter position within the O21 spectral range (filter positions: 1250 to 1293 nm). The use of the exponential fit to the PS luminescence background was empirical. Other fitting models, such as polynomial fits may be used. We observed that the selection of the fit model did not substantially affect the intersample comparison of extracted O21.

A Gaussian curve [Fig. 2(b)] was fit to the background-subtracted spectral data of O21 for the filter positions 1222 to 1315 nm. The full-width at half-maximum bandwidth of the Gaussian fit was ∼25  nm, which is the result of the convolution of the O21 emission feature (∼18  nm) and the spectral bandwidth of the filters (∼15  nm). The O21 quantities used in this work were defined by summing the intensity values of the Gaussian fit for the filter positions 1250 to 1293 nm.

### 2.3 Measurements of ^1^O_2_ in BPD Solutions

The O21 dosimeter system was initially tested using liquid PS solutions. The solutions were prepared as follows. Verteporfin-related compound A (USP, Rockville, Maryland) was dissolved at 10  mg/ml (14.189 mM) in dimethyl sulfoxide (Sigma-Aldrich, St. Louis, Missouri). 20 ml solutions of methanol (Fisher Scientific, Fair Lawn, New Jersey) or phosphate-buffered saline (PBS, Corning, Manassas, Virginia) were diluted to 3 or 10  μM. For the experiments, the solutions were contained in a 3.5-ml fused quartz cuvette (CV10Q3500, Thorlabs).

**Fig. 3:**
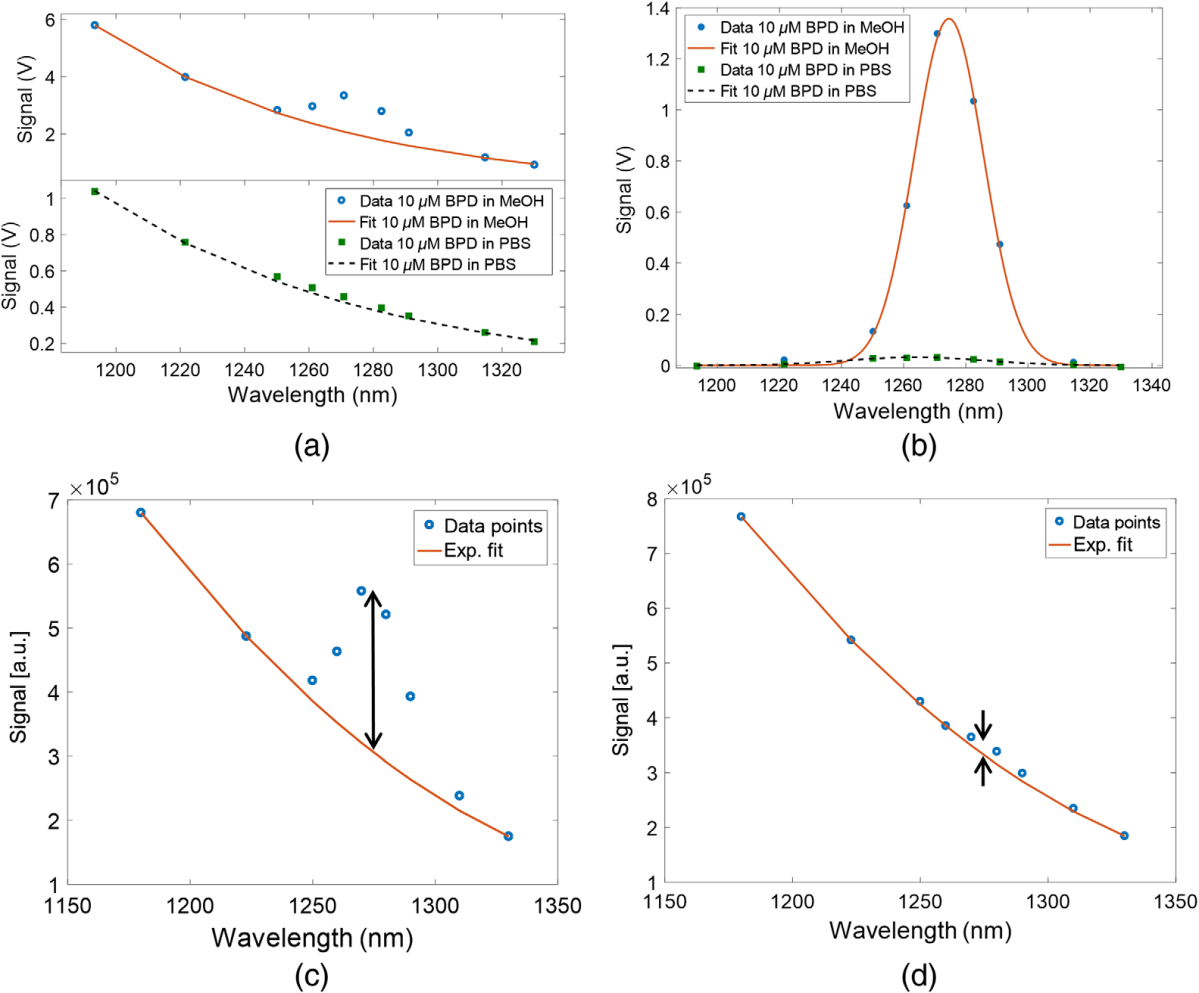
Measurements in PS solutions: (a) raw spectra collected from 10  μM BPD in methanol (top) and PBS (bottom) solutions. (b) Singlet oxygen signal from 10  μM BPD in methanol and PBS solution. (c) Combined BPD luminescence spectra (red line, exponential fit and blue circles, measured data points) from 10  μM BPD in methanol, before nitrogen bubbling. Amplitude of singlet oxygen signal (black double arrow). (d) Combined BPD luminescence spectra (red line, exponential fit and blue circles, measured data points) from 10  μM BPD in methanol, afer 6 min nitrogen bubbling. Amplitude of singlet oxygen signal (black double arrow).

Figure 3(a) shows the raw spectra collected from 10-μM BPD in methanol (top) and PBS (bottom) solutions. The extracted O21 from these two measurements are shown in Fig. 3(b). As displayed in these plots, the O21 signal in methanol is almost two orders of magnitude stronger than in PBS due to strong quenching in PBS. The PS signal in PBS is also somewhat weaker, due to aggregation of the hydrophobic BPD.^32^ The O21 measurements of BPD dissolved in methanol provided the stronger singlet oxygen signal and was used to optimize the dosimeter system. The BPD-PBS solution is a closer representation of the bio- and photochemical environment in tumors. The small O21 to PS ratio in the signal from BPD-PBS indicates the importance of reliable fitting of the PS background, which requires multiple spectral data points.

Spectra were also compared for oxygenated and deoxygenated BPD methanol solutions to verify that the phosphorescence peak at 1270 nm originates from singlet oxygen. Deoxygenation was achieved by bubbling nitrogen gas for 6 min through a 10-μM BPD methanol solution in order to create a deoxygenated luminescence environment. Figures 3(c) and 3(d) show (PS+O21) luminescence/phosphorescence spectra for oxygenated and deoxygenated conditions, respectively. The black double arrows indicate the O21 signal strength generated in both environments. Although simple bubbling of $N_{2}$ does not replace all of the dissolved oxygen, the O21 signal was reduced by an order of magnitude after 6 min of nitrogen bubbling, verifying that the spectral feature centered at 1270 nm was indeed due to O21 phosphorescence.

### 2.4 Animal Model

All animal procedures were approved by the Dartmouth Institutional Animal Care and Use Committee (IACUC), and the protocol was followed as approved in the experiments. A cohort of 19 mice was used in this study. The animal model was developed by inoculation of FaDu cancer cells into the flank of 6- to 8-week old female Athymic nude mice (Charles River/NCI, Bethesda, Maryland). FaDu is a human head and neck carcinoma (ATCC, Manassas, Virginia) cell line.^33^ Fadu cells were cultured in MEM (Hyclone, Logan, Utah) 10% (v/v) fetal bovine serum (Hyclone, Logan, Utah) and 100  IU/mL penicillin–streptomycin (Hyclone, Logan, Utah). Cells were grown in a humidified, 5% ${\mathrm{CO}}_{2}$, 37°C incubator. A total of $1\times 10^{6}$ FaDu cells were used in a 1:1 mixture of media and matigel (BD Biosciences, San Jose, California) injected subcutaneously in a 200-μl volume. Mice were placed on a mouse purified low chlorophyll diet (MP Biomedical, Solon, Ohio) to decrease background autofluorescence. The tumors were allowed to grow for several days and tumor volume measurements were recorded daily using a caliper. Treatment of mice started when tumor volumes met the acceptance criteria: the day 1 (1 day before PDT treatment) tumor volume was 50 to $125\;{\mathrm{mm}}^{3}$ and the day 0 (treatment day) tumor volume was $75\text{-}150\;{\mathrm{mm}}^{3}$, where day 0 was defined as the PDT treatment date.

### 2.5 PDT Treatment

For PDT, Verteporfin (pharmaceutical name for BPD “a”) was injected into the mice. During this process of treatment, mice were kept in a surgical cradle using isoflurane (3% for induction, 1% to 3% for procedure) and the oxygen flow rate was 1 to 2  l/min. A toe pinch was used to confirm that complete anesthesia was present, and mice were closely monitored for depth of anesthesia throughout treatment. PDT light treatment was performed on two groups of mice (treatment and control). Treated mice were first injected with 4-mg/kg Verteporfin (BPD), (USP, Rockville, Maryland) via tail vein, and allowed to wake and incubate the Verteporfin for 1 h. BPD is not selectively accumulated in tumors, rather it is brought in through the neovascular leakage and lack of lymphatic clearance, resulting in a gradual build up in the tumor, as occurs with most porphyrins. Steady-state detection measures all generated singlet oxygen, but there is known to be a reasonably high fraction in the tumor tissue relative to the vasculature after 1 h of incubation.^34^^,^^35^ After this incubation time, the mice were again put into the surgical cradle and given a light treatment using a 690-nm laser at a laser intensity of $200\;\mathrm{mW}/{\mathrm{cm}}^{2}$ (1-cm laser spot diameter) for a duration of 1000 s ($200\;J/{\mathrm{cm}}^{2}$). Mice were allowed to recover and tumor sizes were recorded daily, using a caliper to measure the tumor volume, until humane endpoints were reached according to the approved IACUC protocol. The procedure for the control group was identical to the treatment group with the difference that mice in the control group did not receive a Verteporfin injection.

The tumor sizes of all 19 mice were measured daily following injection with tumor cells. The growth curves shown in Sec. 3.1 include 5 days before and 5 days after PDT treatment. These growth curves were plotted using tumor volumes versus time (days with respect to the treatment date of day 0). They were also plotted on a semilogarithmic scale to determine the growth rates before and after the treatment. Growth rates were estimated by fitting an exponential trend line to the growth curves (being linear on a semilog graph), carrying the unit ${\text{day}}^{-1}$. The tumor growth inhibition was correlated to the amount of O21 generated during the treatment. For that purpose, the relative change in growth rate was plotted against the measured O21 values. The relative change in growth rate was defined as: (growth rate after treatment/growth rate before treatment).

### 2.6 ^1^O_2_ Dosimetry Measurements During PDT of Mice

The combined O21/PS phosphorescence/luminescence was measured as described in Sec. 2.2. The measurement was performed during the treatment for all 19 mice. Each PS/O21 spectrum was measured in 54 s and repeated 14 times during the 1000-s runs, which was the entire treatment period for each mouse. The quantitative value for each measurement was determined as discussed in Sec. 2.2. The average value of these 14 measurements was used to determine the O21 and PS values for each mouse.

### 2.7 Statistical Analysis

The measured O21 and PS values were analyzed and tested for correlation with the fitted tumor growth inhibition values post-treatment versus pretreatment, using both the light-only control animals and the PDT-treated animals. The light only controls provided values for near zero O21 and limited growth inhibition, and a students’ t-test was used to assess the global difference in O21 produced and PS present in the PDT versus control groups. A standard Pearson’s correlation coefficient was calculated with respect to these two correlation tests, and $R^{2}$ values were evaluated.

## 3 Animal Study Results and Discussion

### 3.1 Tumor Growth Inhibition after PDT Treatment

**Fig. 4:**
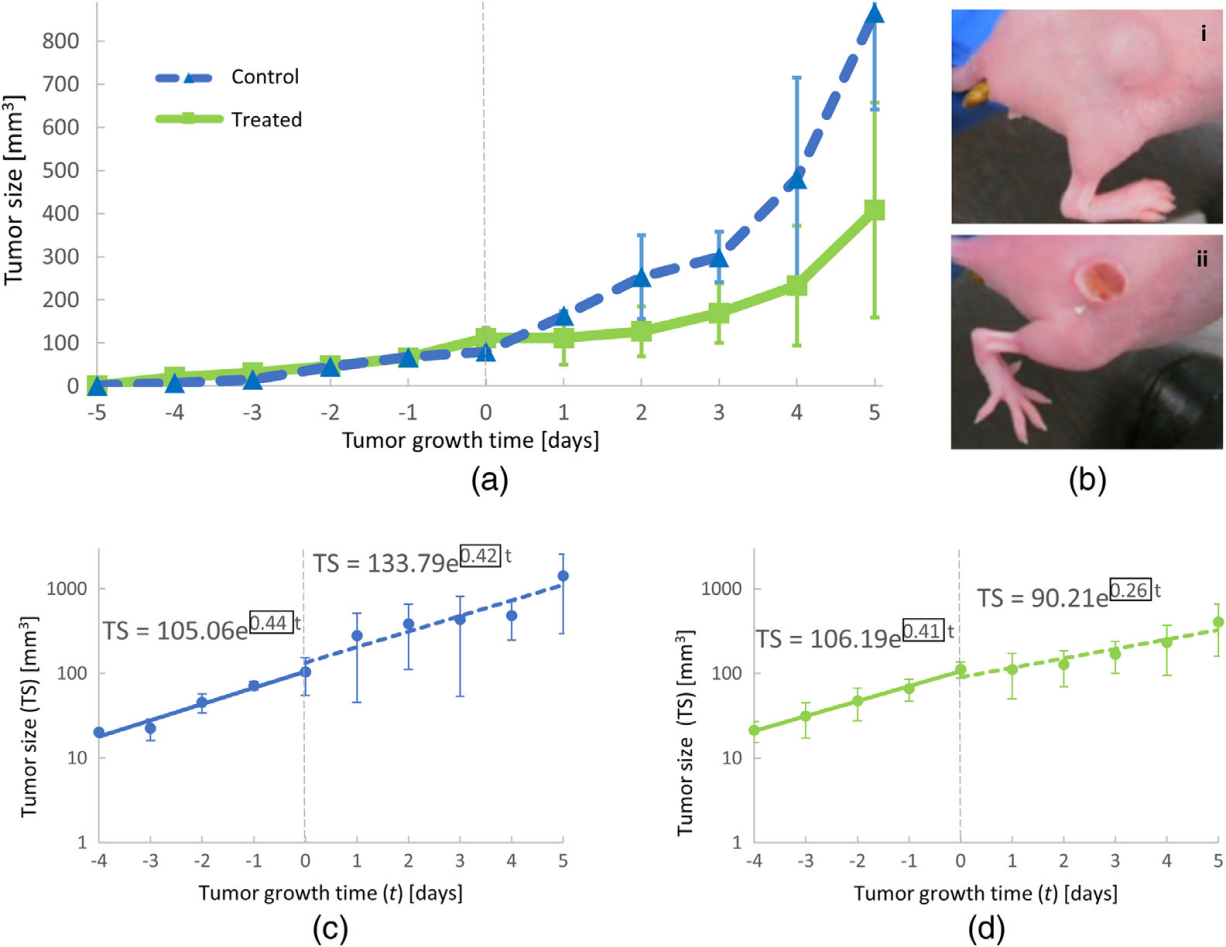
Inhibition of tumor growth following PDT treatment of mice: (a) average tumor growth curves for control (blue) and treated (green) mice observed before (time < day 0) and after (time > day 0) PDT treatment. The standard deviations for the measurements are indicated by error bars. (b) Example photographs of (i) control and (ii) treated tumors 3 days after PDT. (c) Comparison of average tumor growth rates (black boxes) before (solid, blue trend line) and after (dashed, blue trend line) light exposure for control mice. (d) Comparison of average tumor growth rates (black boxes) before (solid, green trend line) and after (dashed, green trend line) PDT treatment for treated mice.

Tumor growth curves were recorded for all mice using the method described in Sec. 2.5. Figure 4(a) shows the average tumor growth behavior for control and treated mice. In general, similar growth was observed for all mice before PDT treatment on day 0. After PDT treatment, the tumor growth was faster for control mice compared with the growth of the treatment group. An example of visual changes to treated tumors is shown in Fig. 4(b). The top panel Fig. 4b (i) shows a tumor in a control mouse 3 days after light exposure. Clear visual changes to a treated tumor (3 days after light exposure) are visible in the bottom panel Fig. 4(b) (ii). A quantitative comparison of average tumor growth before (time < 0 days) and after PDT treatment (time > 0 days) is shown for control [Fig. 4(c)] and treated [Fig. 4(d)] mice. For control mice, the average growth rate remained within 5% of its prelight exposure value, changing from 0.44 (blue solid line) to $0.42\;{\text{day}}^{-1}$ (blue dotted line) after light exposure [Fig. 4(c)]. In contrast, PDT treatment led to a reduction in average growth rate by ∼40% [Fig. 4(d)], decreasing from 0.41 (solid green line) to $0.26\;{\text{day}}^{-1}$ (dotted green line). These results show a clear effect of the PDT treatment on the tumor tissue. Within 5 days after light exposure, the average tumor size of control mice was twice as large as the tumor size of treated mice. The growth rate of tumors before PDT treatment was similar to the growth rate of control mice, verifying that the decrease in tumor growth rate was induced by the PDT treatment.

### 3.2 ^1^O_2_ and PS Quantitation During PDT Treatment

**Fig. 5:**
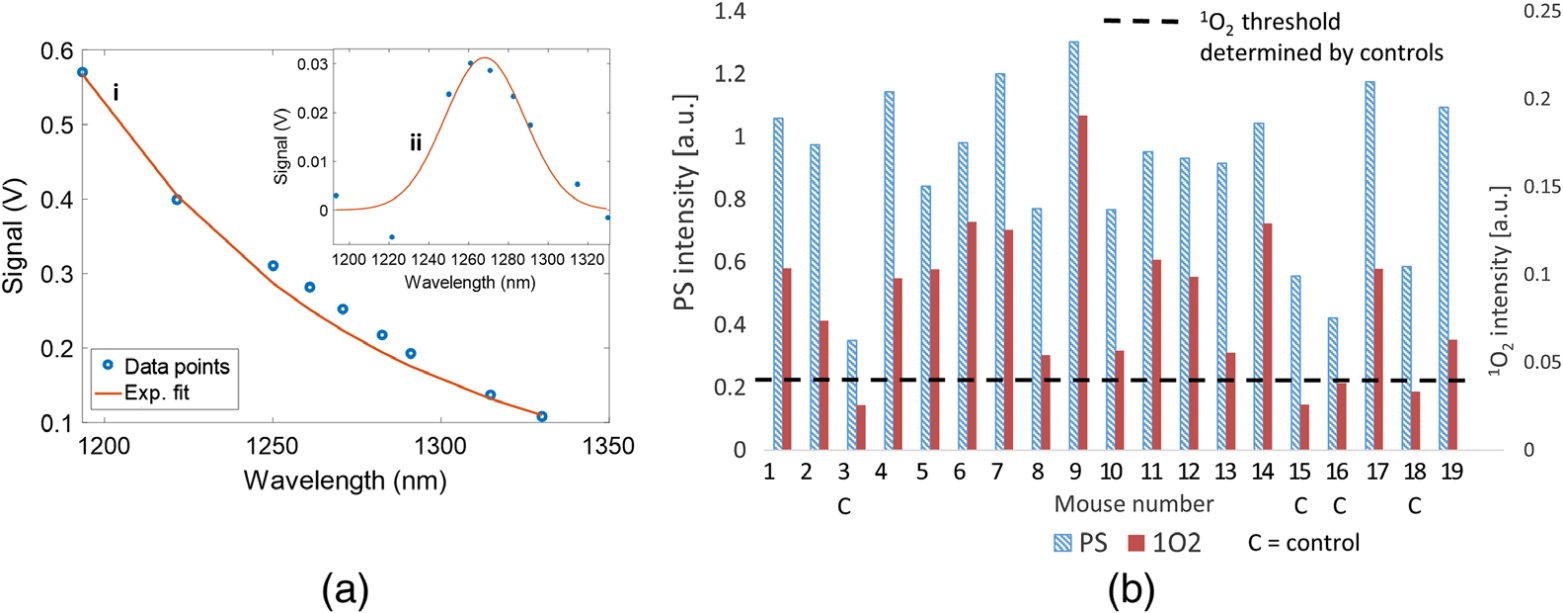
Quantitation of generated O21 during PDT treatment of mice: (a) example (mouse #1) of the convoluted PS+O21 spectrum (i) recorded during PDT treatment. The inset (ii) shows the extracted O21 signal fitted with a Gaussian curve. (b) Measured singlet oxygen (brown) and PS (blue shaded) signals during PDT treatment of 19 mice. Dotted black line indicates O21 threshold measured in control mice, which are indicated with a “c” under the x-axis.

Figure 5(a) shows an example (mouse #1) of the convolved PS+O21 spectrum (i) recorded during PDT treatment. The inset (ii) in Fig. 5(a) shows the extracted O21 signal fitted with a Gaussian peak curve. The measured O21 (solid brown) and PS (shaded blue) phosphorescence/luminescence values for all mice are shown in Fig. 5(b). The distribution of measured O21 and PS values shows lower O21 and PS in all control mice as compared with the treated mice. The PS and O21 signals measured in control mice are due to the photosensitizing processes of the endogenous fluorophores in the skin such as porphyrins.

**Fig. 6:**
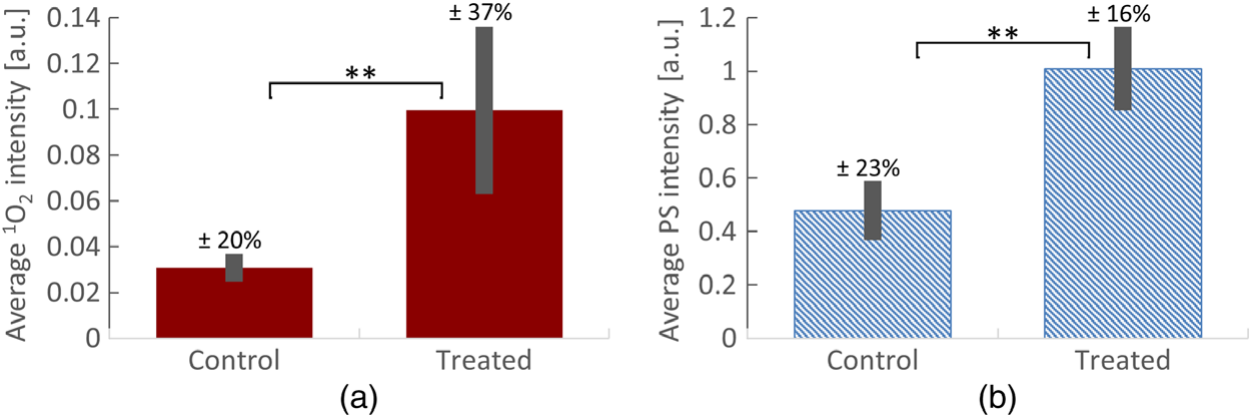
Comparison of generated O21 and PS luminescence during PDT treatment of mice: (a) average measured O21 signal for (left) control and (right) treated mice. Signals are significantly different with p-value<0.005. The O21 signal for control and treated mice has a variability of ±20% and ±37%, respectively. (b) Average measured PS signal for (left) control and (right) treated mice. Signals are significantly different with p-value<0.005. The PS signal for control and treated mice has a variability of ±23% and ±16%, respectively.

Figures 6(a) and 6(b) show measured average values (from all 19 mice) of O21 phosphorescence and PS luminescence, respectively. The measured average (all 19 mice) O21 values are higher for treated mice than for control mice [Fig. 6(a)], with a two star level of significance (p-value<0.005). The same level of statistical significance (p-value<0.005) was found for the measured average PS values for control and treated mice [Fig. 6(b)]. The statistical significance was estimated using two-tailed students’ T test as described in Sec. 2.7.

For the treated groups, the variability of the measured O21 (±37%) [Fig. 6(a), right] is approximately twice as high as the variability in the measured PS signal [±16%, Fig. 6(b), right]. However, for the control group, the variabilities in signal are similar for O21 (±20%) and PS (±23%), as shown in Fig. 6(a), left and Fig. 6(b), left, respectively. We attribute the higher variability in measured O21 of treated mice to variations in biological and biochemical parameters between individual mice and their tumors, such as local oxygen concentration. As discussed later, this is also supported by the correlation of O21 with relative change in tumor growth rate (Fig. 8). Note that the oxygen concentration is not reflected in the PS signals, which do not show these high variations.

### 3.3 Temporal Evolution of ^1^O_2_ and PS

**Fig. 7:**
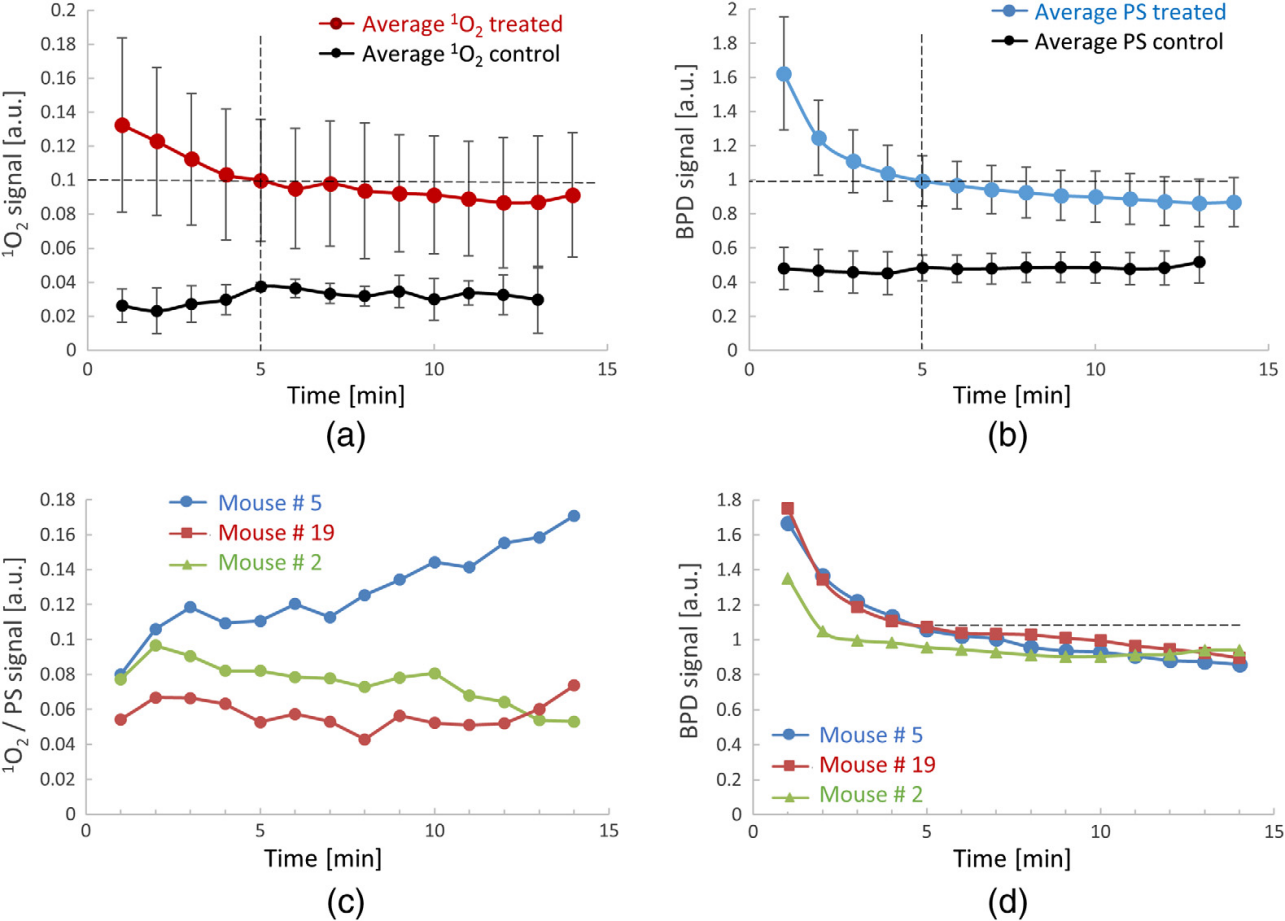
Time trends of generated O21 during PDT treatment of mice: (a) measured average singlet oxygen signal during PDT treatment of of 19 mice (brown). The measured average singlet oxygen signals for control mice are shown in black. The standard deviations of the measurements are indicated by error bars. (b) Measured average PS signal during PDT treatment of mice (n=19) (blue) and control mice (black). The standard deviations of the measurements are indicated by error bars. (c) Example time trends (mouse # 5, 19, 2) of O21 signal during PDT treatment. (d) Example time trends (mouse # 5, 19, 2) of PS (BPD) signal during PDT treatment. Dotted lines show the trends.

Figures 7(a) and 7(b) show the average time trends (from all 19 mice) of measured O21 and PS, respectively. The average temporal trends for treated and control mice are plotted separately. Figures 7(c) and 7(d) show temporal evolutions of O21 and PS signals of three individual mice. The O21 temporal trends in Fig. 7(c) are plotted as the ratio of O21/PS, which provides additional information about the correlation between the O21 and PS signal time trends. The dotted lines in the graphs are visual aids for trend evaluation. Both the PS [Fig. 7(b)] and O21 signals decrease monotonically through the process of photobleaching. A slower O21 decrease is visible during the first 5 min of PDT treatment, followed by a partial recovery of the signal. The time trends in Figs. 7(c) and 7(d) show that both the PS and O21 signals decrease over time but the ratio of O21 to PS stays relatively invariant. Larger mouse to mouse variations in O21 than in PS are observed, evident by the large standard deviation [error bars in Fig. 7(a)] and by the difference of individual traces in Fig. 7(c), compared to those in Figs. 7(b) and 7(d), respectively. The strong variability in the O21 signal is attributed to biological heterogeneity and differences in the ability to generate O21 in different tumor environments (e.g., oxygenation concentration) for different mice.

### 3.4 Correlation of Relative Change in Tumor Growth Rate with Generated ^1^O_2_

**Fig. 8:**
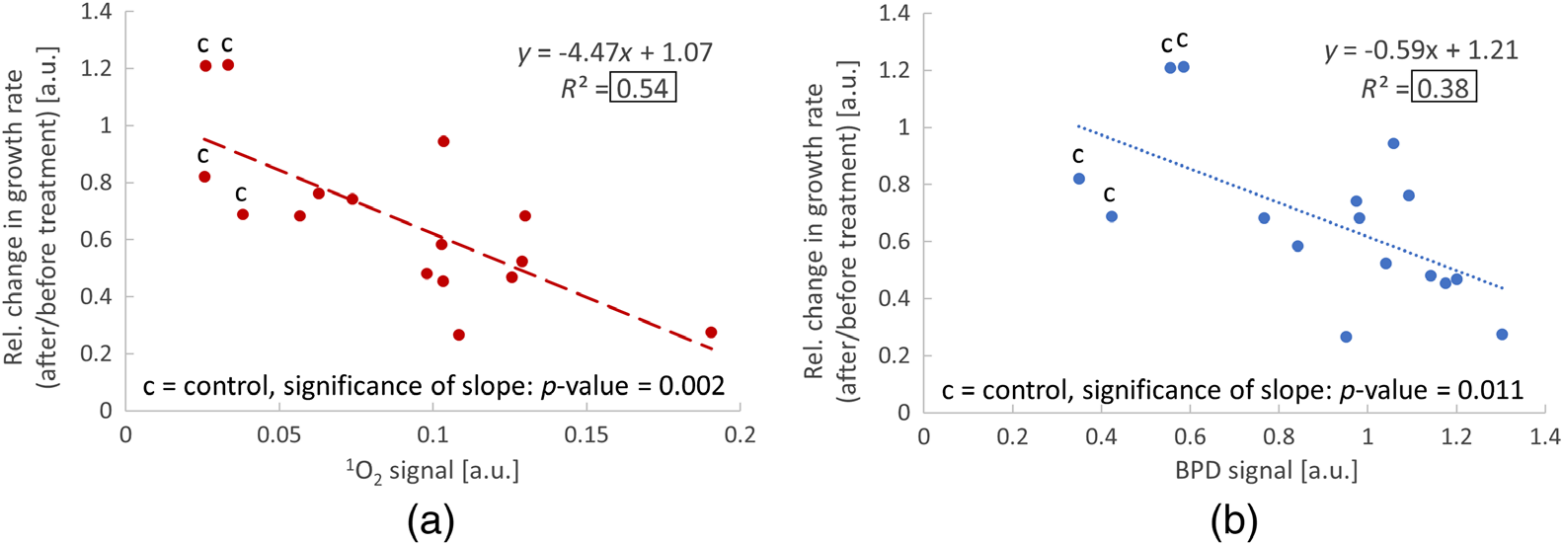
Correlation of PDT treatment efficiency to the generated singlet oxygen and PS: (a) correlation of relative change in tumor growth rate (ratio of growth rates after/before PDT treatment) to measured singlet oxygen amount for all mice. (b) Correlation of relative change in tumor growth rate to measured PS amount for all mice (n=19). In both plots, “c” indicates control mice.

The plots in Figs. 8(a) and 8(b) show the correlations of relative change in tumor growth rate to the amount of O21 and PS measured from all 19 mice during PDT treatment, respectively. The relative change in growth rate was determined for each mouse following the method described in Sec. 2.5 (ratio of growth rates after/before PDT treatment). Data points of control mice are indicated in the plots by the letter “c.” We note that the PS and O21 signals measured in control mice are due to the photosensitizing processes of the endogenous fluorophores in the skin. Linear regression curves (dotted lines) were fitted to both data sets resulting in negative slopes and a goodness of fit of $R^{2}=0.54$ and $R^{2}=0.38$ for O21 [Fig. 8(a)] and PS only [Fig. 8(b)], respectively. The $R^{2}$ values are highlighted in the plots by black boxes. In addition, p-values were calculated to verify that the slopes of the regression lines are significantly different from a slope of zero. The calculated values are p-value=0.002 and p-value=0.011 for O21 and PS, respectively. Both p-values are smaller than 0.05 indicating a significant correlation.

Data in Fig. 8(a) show a linear correlation between the relative change in tumor growth rate and the amount of O21 that was generated during PDT treatment. The negative slope of the regression curves implies that higher amounts of generated O21 lead to a stronger inhibition of tumor growth. This correlation follows the model that the oxidative stress induced by O21 is an important mechanism of tumor cell death during PDT. Figure 8(b) shows a much weaker correlation with the measured PS signal. Since the production of O21 depends on the amount of excited PS, some correlation is expected. However, as discussed above, O21 depends on additional factors including tissue oxygenation that cannot be monitored by PS luminescence. A comparison of the linear regression curves in Figs. 8(a) and 8(b) demonstrates that the relative change in tumor growth rate has a stronger correlation to O21 than to the PS signal. This further explains the heterogeneity of tumor growth curves after PDT treatment and the large variability in measured O21 that was observed in Figs. 6 and 7. This finding indicates that the tumor response to PDT treatment does not simply depend on the administered light and drug dose but rather on the tumor’s biochemical environment and the ability to generate O21 through the interaction of the treatment laser with the PS.

## 4 Conclusions

In this study, a new dosimeter system was demonstrated that could simultaneously measure PS and singlet oxygen generated directly by a cw PDT light source with high SNR and low crosstalk. To our knowledge, this is the first time that O21 was quantified during *in vivo* PDT using a cw treatment laser source with multispectral fitting of the signal. Since cw sources are primarily used in current clinical PDT treatment protocols, this cw O21 dosimeter instrument can be incorporated into existing PDT systems. The relevance of quantitating O21 was demonstrated by successfully correlating the measured O21 to the relative change in tumor growth rate. Even though all animals used in this study had similar tumor volumes and received the same injected PS doses and light doses, variations of up to 37% were seen in tumor volume regrowth and singlet oxygen produced. However, the correlation between singlet oxygen and tumor regrowth delay appears to indicate that there is predictive value in this dosimetry measurement. The signal is exceptionally low in intensity, but through amplified detection and spectral fitting, a robust measurement was achieved *in vivo*. Future work will develop a more comprehensive database to further demonstrate the correlation of PDT outcome to the O21 generated. The clinical goal is to be able to adjust the light dose during PDT treatment in real time to minimize variations in treatment success between subjects.
